# Effect of Wax Additives and Silanization of Diatom Surfaces on Thermomechanical Properties of Polylactide Composites

**DOI:** 10.3390/polym14245511

**Published:** 2022-12-16

**Authors:** Marta Dobrosielska, Renata Dobrucka, Martyna Pajewska-Szmyt, Paulina Kozera, Ewa Gabriel, Julia Głowacka, Dariusz Brząkalski, Krzysztof J. Kurzydłowski, Robert E. Przekop

**Affiliations:** 1 Faculty of Materials Science and Engineering, Warsaw University of Technology, ul. Wołoska 141, 02-507 Warsaw, Poland; 2 Department of Non-Food Products Quality and Packaging Development, Institute of Quality Science, Poznań University of Economics and Business, al. Niepodległości 10, 61-875 Poznań, Poland; 3 Centre for Advanced Technologies, Adam Mickiewicz University in Poznań, ul. Uniwersytetu Poznańskiego 10, 61-614 Poznan, Poland; 4 Faculty of Chemistry, Adam Mickiewicz University in Poznań, ul. Uniwersytetu Poznańskiego 8, 61-614 Poznań, Poland; 5 Faculty of Mechanical Engineering, Bialystok University of Technology, ul. Wiejska 45c, 15-351 Bialystok, Poland

**Keywords:** polylactide, diatomaceous earth, beeswax, diatomite, silanes, chemical modification, mechanical properties, PLA

## Abstract

In the present study, tests were conducted on high-filled composite samples on a polylactide matrix, modified with diatomaceous earth, three types of silanes, and natural and synthetic wax. The obtained samples were characterized in terms of the effect of modifications on mechanical properties (tensile strength, flexural strength, and impact resistance) or processing properties, e.g., melt flow rate (MFR). The study showed that the modification had a favorable effect on the processing properties of the composites, associated with up to an eight-fold increase in flow rate index compared with the reference sample, especially for samples treated with methyltrimethoxysilane (MTMOS), and up to a ten-fold increase under low shear-rate flow conditions. The effect of the addition of waxes of different origins (synthetic and natural) was also determined, and it was shown that beeswax tended to reduce the flow rate of the composites regardless of the silane used. The addition of synthetic wax to composites increased the tendency to agglomerate diatomaceous earth, while natural wax had a positive effect on filler dispersion.

## 1. Introduction

Today, due to ubiquitous environmental pollution, there is an increasing emphasis on producing more goods from renewable raw materials, decreasing carbon emissions, and increasing the biodegradability or recyclability of different types of products. We can also see a clear shift away from the production of materials that use petroleum-based compounds. Additionally, generating large amounts of waste is undesirable from an environmental point of view. Therefore, new environmentally friendly solutions are constantly being sought. Polylactide (PLA) has a number of advantages and in many cases can be used to replace petroleum-based polymers due to its good physicochemical and mechanical properties. Various types of fillers, including fillers of natural origin such as diatomaceous earth [1,2,3], cellulose fibers [4,5], and even wood flour/fibers [6,7], are used as modifiers of the physicochemical properties of PLA. They can also be used to modify the properties of a range of other polymers. Mineral fillers such as talc, kaolinite [8], montmorillonite [9,10], hydroxyapatite [11,12], fumed silica (aerosil), bentonite, and mica [13] are also used. This makes it possible to obtain different composite properties depending on the desired purpose. Often, natural fillers tend to agglomerate in the polymer matrix, and therefore the use of dispersion-enhancing additives can favorably affect the mechanical properties of composites as the problems of early cracking or low-energy crack propagation are limited or eliminated. Positive effects of the silanization of fillers resulting in improved dispersion in the matrix are described in the literature. Silane coupling agents are used for the modification of inorganic compounds as they are capable of reacting with surface hydroxyl groups and contain various types of functional groups in their structure that determine the chemical compatibility of the modified surface with the polymer matrix, as well as the mechanism of its interaction with the matrix [14]. Surface silanization is a conventional and low-cost method of improving the interfacial interaction between the filler surface and the polymer matrix. Silane coupling agents act as adhesion promoters and reduce the tendency of fillers to form agglomerates in the polymer matrix. They have the ability to form covalent bonds between organic and inorganic materials in some cases. During the modification process, the hydrolysed silane modifier converts to silanol and reacts with hydroxyl groups on the material’s surface to form strong hydrogen bonds [15]. Kucuk et al. described the effect of silane coupling agents with different functional groups on the properties of thermoplastic polyurethane/diatomaceous earth (TPU/DE) composites. The surface modification of diatomaceous earth, among other procedures applied, improved mechanical properties and reduced the formation of filler agglomerates. The authors schematically presented interfacial interactions via silane surface modifiers between the mineral filler and the TPU [16]. The addition of natural fillers, including diatomaceous earth or various types of sludge or fibers, especially in high concentrations, can adversely affect the processing and performance properties of composites, including processing rheology and mechanical properties. In order to eliminate these effects, it is advantageous to use processing additives, primarily, from the ecological point of view, agents of natural origin, which not only have a positive effect on the processing properties but can also help increase certain mechanical and physicochemical parameters. Typical processing additives of natural origin are waxes and oils of plant or animal origin and their semi-synthetic derivatives. Various types of waxes are used for this purpose, including jojoba oil-wax [17], mango seed wax [18], paraffin wax [19], epoxidized jatropha oil [20], epoxidized soybean oil methyl ester [21], and maleinized linseed oil [22]. In previous work [23], it was shown that the particle size of diatomaceous earth used as a filler for PLA matrix composites has a significant effect on the mechanical properties of the obtained materials. The smaller the particle size, the higher the impact resistance of the composite, while as the particle size increases, the mechanical properties deteriorate. In the present study, PLA-based composites with processing additives in the form of natural and synthetic waxes were also tested. However, the filler, i.e., base diatomaceous earth containing a full cross-section of diatoms of different sizes, was pre-silanized. The effect of the addition of waxes in varying amounts on the properties of the produced composites, especially their processing properties and degradation rates, was investigated. The dispersion of the filler in the polymer matrix was also determined in terms of the differences caused by the addition of waxes of different origins. 

## 2. Materials and Methods

### 2.1. Materials

PLA, type Ingeo 4043D, was purchased from NatureWorks (Minnetonka, Minneapolis, MN, USA). Diatomaceous earth (Perma-Guard, Bountiful, UT, USA) was derived from diatomite deposits. Synthetic wax WTH-B microbeads (behenamide, C_22_H_45_NO) were purchased from WTH GmbH (Hamburg, Germany). Beeswax was received from APIS Apiculture Cooperative (Lublin, Poland). n-octyltriethoxysilane (OTES), 3-glycidoxypropyltrimethoxysilane (GPTMOS), and methyltrimethoxysilane (MTMOS) were purchased from Sigma-Aldrich (Saint Louis, MO, USA).

### 2.2. Preparation of Modified Diatomaceous Earth

**Figure 1:**
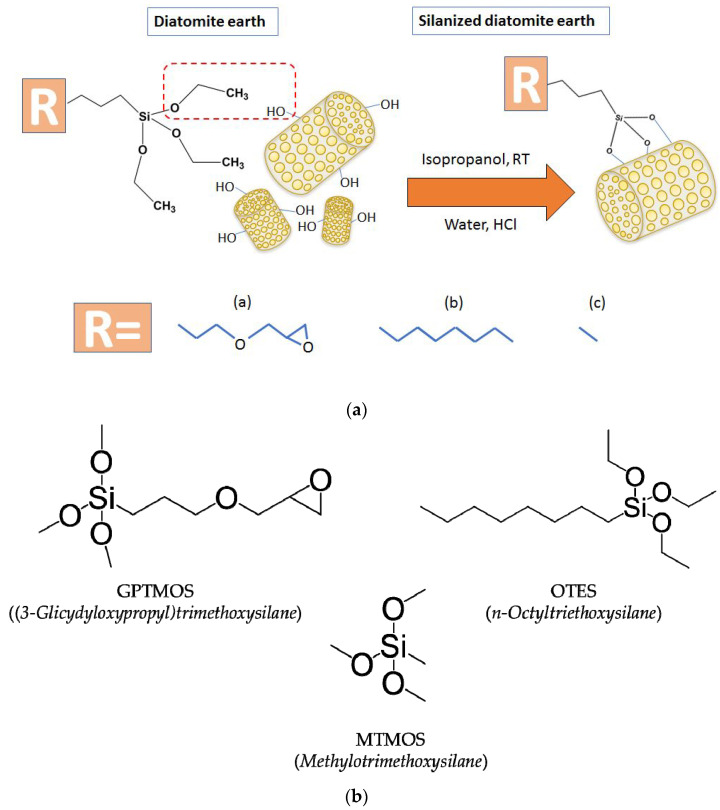
(**a**) Silanization process. (**b**) Structural formulas of organofunctional silanes used to modify diatomaceous earth.

1 kg of diatomaceous earth was placed in a 20 L glass reactor equipped with a mechanical stirrer. Then, 4 L of iPrOH, 10 mL of demineralized water, and 1% *w*/*w* of the appropriate silane (GPTMOS, OTES, or MTMOS) were added to the reactor and left for two hours with constant stirring (300 rpm). After 2 h, 10 mL of concentrated hydrochloric acid (HCl) was added in portions. After 24 h of continuous mixing, the mixture was transferred to a separate container and allowed to settle. After the liquid was decanted from the precipitate, the diatomaceous earth was washed with demineralized water and placed in an oven at 60 °C for 24 h. The silane formulas are shown in Figure 1a and a scheme of the silanization process is shown in Figure 1b.

### 2.3. Preparation of Granulates

PLA-based composites were prepared using a ZAMAK MERCATOR WG 150/280 (Zamak Mercator, Skawina, Poland) laboratory two-roll mill. For this purpose, 250 g of PLA 4043D was mixed at 210 °C together with 250 g of modified or base diatomaceous earth and 2% or 4% by weight of natural (beeswax) or synthetic (WTH-B) wax, respectively. The process was carried out until homogeneous mixtures were obtained. The resulting systems were then ground using a WANNER C17.26 sv mill and dried for 24 h at 60 °C. Analogous systems containing unmodified diatomaceous earth were also prepared. 

### 2.4. Preparation of Final Samples

The finished masterbatches were diluted 1:1 with PLA directly on an Engel e-victory 170/80 injection molding machine. Table 1 shows the injection molding parameters. A holding pressure with a linear increment over time was applied. The mold temperature was maintained at 80 °C. Standardized specimens for mechanical testing in accordance with PN-EN ISO 20753:2019-01 were fabricated. The final concentrations of the systems, together with sample symbols, are shown in Table 2.

### 2.5. Characterization Methods

For flexural and tensile strength tests, the materials obtained were cut down into type 1B bar specimens in accordance with EN ISO 527:2012 and EN ISO 178:2006. Tests of the obtained specimens were performed on an INSTRON 5969 universal testing machine with a maximum load force of 50 kN. The traverse speed for tensile strength measurements was set at 2 mm/min, and for flexural strength it was also set at 2 mm/min. A Charpy impact test (with no notch) was performed on an Instron Ceast 9050 impact machine (Instron, Norwood, MA, USA) according to ISO 179−1: 2010 Plastics−Determination of Charpy impact properties—Part 1: Non-instrumented impact test, PKN, 2010. The melt flow rate (MFR) was measured using the Instron CEAST MF20 melt flow indexer (Instron, Norwood, MA, USA), which accords with EN ISO 1133, at 210 °C for a load of 2.16 kg. The thermal properties of the materials (T_g_, T_cc_, T_m_) were studied using a Q1000 differential scanningg calorimeter (TA Instruments, New Castle, DE, USA). Samples with a weight of 8.0 + 0.2 mg were placed into an aluminum hermetic pan. First, the samples were equilibrated at −90 °C, heated to 230 °C with a scan rate of 10 °C/min, and cooled to −90 °C with a scan rate of 10 °C/min. Finally, they were again heated to 270 °C with a scan rate of 10 °C/min. The process was conducted in a nitrogen atmosphere. Using Universal V4.5A TA software, the glass transition temperature (T_g_) was determined as the midpoint of the glass transition temperature range. The cold crystallization temperature (T_cc_) and melting temperature (T_m_) were taken as the peak temperatures of the cold crystallization and melting, respectively. Dynamic mechanical analysis (DMA) was performed using a Q800 DMA (TA Instruments, New Castle, DE, USA) in a dual cantilever mode that accords with ASTM D4065−01. From each composite, three rectangular specimens 60 mm long and 10 mm wide were cut and used in the testing. The analysis was conducted from 0 °C to 130 °C, with a heating rate of 3 °C/min at a frequency of 1 Hz and amplitude of 30 µm. The glass transition temperature (T_g_) was determined from the storage modulus curve. The size of the diatoms used to prepare the composites was measured with a Mastersizer 3000 (Malvern Instruments Ltd., Malvern, UK). The measurements were made for the samples in water suspension (Hydro EV attachment). For the wet samples, the stirrer revolution speed was 2330 RPM and the ultrasound power was 70%. The FT-IR measurements were carried out using a Thermo Scientific Nicolet iS50FT-IR spectrometer in transmission mode. Samples weighing 4 mg were mixed with 200 mg KBr and pressed at 10 tons into the form of discs. The spectra were recorded using eight scans of background measurements and sixteen scans of sample measurements in the 400–4000 cm^−1^ range using a DTGS detector and a KBr beam splitter at 0.5 nm resolution 

## 3. Results and Discussion

### 3.1. Characterization of Silane-Modified Diatomaceous Earth

**Figure 2:**
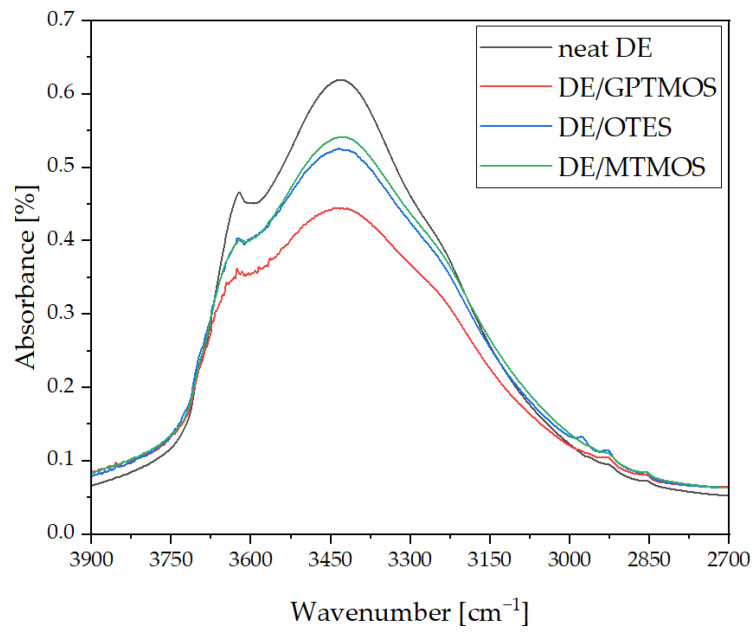
FT-IR spectra of neat diatomaceous earth and silane-modified diatomaceous earth.

FT-IR analysis of the neat diatomaceous earth (DE) and silane-modified diatomaceous earth (DE/OTES, DE/GPTMOS, and DE/MTMOS) was carried out (Figure 2). The analysis showed differences in band intensities after the silanization process. A reduction in the -OH stretching band surface area in the 3000–3700 cm^−1^ range and the appearance of a -CH stretching band in the 2800–3000 cm^−1^ range after the silanization process were observed, with the strongest effect seen for the GPTMOS and similar observations recorded for the other two silanes (OTES and MTMOS). These features confirm the successful silanization reaction of the diatomite surface.

### 3.2. Rheology—Melt Flow Rate (MFR) and Capillary Viscosity

**Figure 3:**
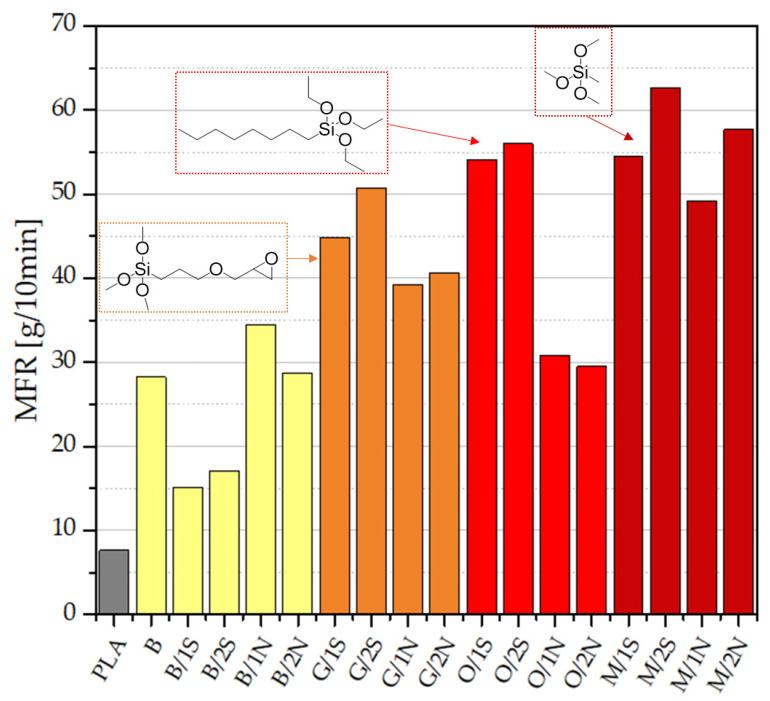
Melt flow rate of granules after the injection process.

Neat, unmodified PLA has a melt flow rate (MFR) of 7.6 g/10 min, which is consistent with reports in the literature [24]. Modification of the PLA with diatomaceous earth in each case resulted in a significant improvement in the processing parameters, which was also observed in our previous work [23] (Figure 3). The positive effect of the addition of waxes on the magnitude of the MFR parameter is clearly visible—as little as a 1% addition of synthetic wax resulted in a two-fold increase in MFR (B/1S). The highest result for the composite containing unmodified diatomaceous earth was achieved for a 1% concentration of beeswax (B/1S, MFR = 34.5 g/10 min). Further increases in the concentration of wax in the system resulted in a slight, in most cases further, increase in MFR values. Preparation of samples of composites containing silane-modified diatomaceous earth (GPTMOS, MTMOS, and OTES) produced a positive effect on the melt flow rate, i.e., MFRs in the range of 29.5–62.7 g/10 min. Modification of the systems with MTMOS resulted in the greatest changes in melt flow rate, reaching values of up to 62.7 g/10 min, which caused minor difficulties in obtaining composites by injection molding. The difference between the MFRs of the O/xS and O/xN systems were due to the effect of the nonpolar octyl-substituted silane displacing the more polar, synthetic wax from the filler to the polymer phase, while the highly apolar synthetic wax (being composed mostly of fatty acid esters of long-chain alcohols [25]) was likely in higher concentration at the filler surface or filler-matrix interphase. On the other hand, more polar methyl-substituted silane in the M/xS and M/xN systems did not result in such behavior to a meaningful extent.

**Figure 4:**
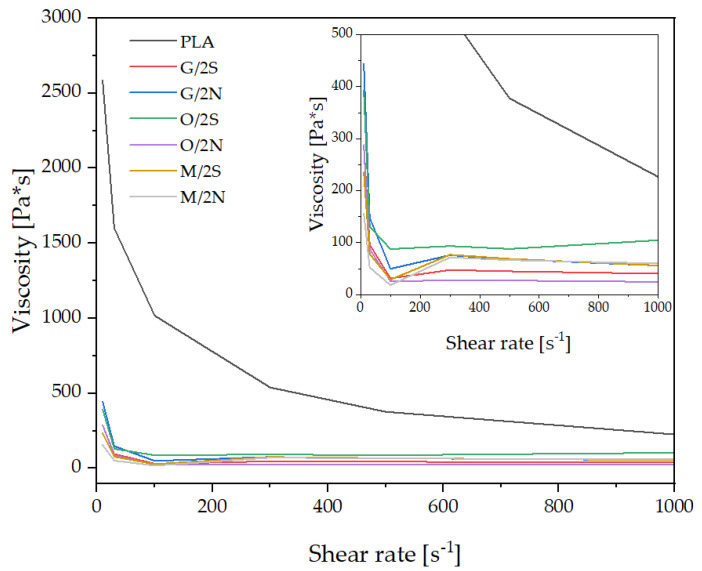
Capillary viscosity of selected composites.

Capillary viscosity tests were conducted for selected composites. The viscosity of the PLA/DE composites modified with silanes was significantly lower than that of the pure PLA (Figure 4) and did not exceed 450 Pa*s independently of the shear rate, with the viscosity decreasing sharply with the increasing shear rate. Once the shear rate reached 100 1/s, viscosity reached a plateau and remained relatively constant for all composites, which was due to the shear thinning effect typical of polymer thermoplastics. The highest viscosity among the composites was recorded for the O/2S system, while the lowest was observed for the O/2N system. It is not clear from the above study which silane additive has the greatest effect on the viscosity of the composites, since the viscosity curves of the produced composites are too similar, but based on this observation it can be stated that the viscosity is both related to the choice of silane and the wax, meaning that there is an interaction between the wax additive and the surface of the silanized filler.

### 3.3. Mechanical Tests

**Figure 5:**
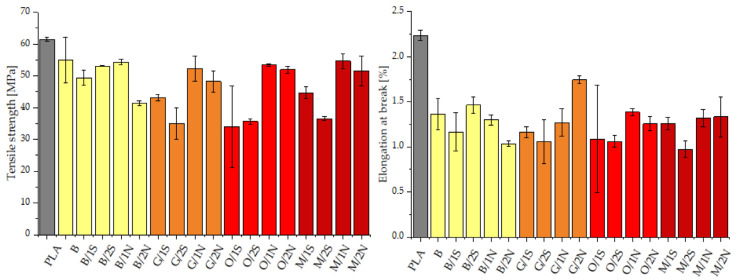
Tensile strength and elongation at break of PLA matrix composites.

Tensile tests carried out on the composites containing diatomaceous earth and silanes showed that the addition of 25% filler resulted in a decrease in both the tensile strength and elongation at break, which was expected due to the properties of diatomaceous earth, the geometry of the filler grains classifying it as a particulate microfiller (Figure 5). Despite this, the decrease in tensile strength was not so drastic (even >80% of the strength of the base PLA is retained), especially for the composites containing natural beeswax. The addition of the wax, especially the natural wax, regardless of concentration, resulted in improved dispersion (lower error bars) of the diatomaceous earth in the PLA. The modification of the diatomaceous earth with the silanes and the simultaneous addition of the synthetic wax, especially at a concentration of 2% by weight, resulted in a definite reduction in tensile strength values below 40 MPa, depending on the modifier. On the other hand, regardless of the type of silane used, its combination with natural wax resulted in the composites being almost as resistant to tensile stress as neat PLA, while improving other parameters, including processing (the aforementioned improvement in melt flow rate and capillary viscosity). The elongation at break was highest for neat PLA due to the continuity of the polymer phase, and its decrease was caused by its discontinuity introduced by the rigid, non-deformable filler grains. In addition, the introduction of diatomite also caused the introduction of air into the plastic, which can further affect crack propagation. The composites modified with the addition of beeswax again showed higher values than those modified with synthetic wax, while the highest elongation at break was recorded for the composite modified with GPTMOS silane and 2% beeswax. 

**Figure 6:**
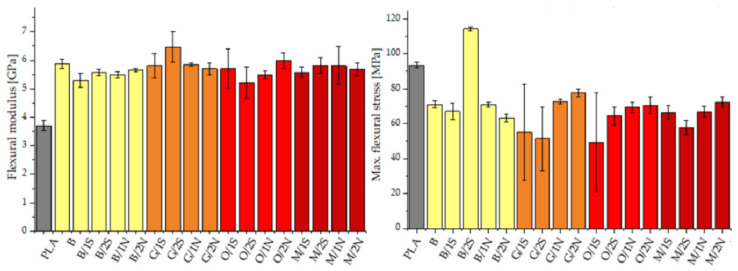
Flexural modulus and maximum flexural stress of PLA matrix composites.

The flexural modulus for the diatomaceous earth-modified composites increased compared with the reference sample, a phenomenon typical of polymer composites (Figure 6). The maximum flexural stress determined for the composites was in almost all cases noticeably lower than that of neat PLA, for similar reasons as those relating to the static tensile tests. For the silane-modified systems, the highest values were recorded for samples with added synthetic wax, especially at higher concentrations, while the addition of natural wax in combination with silanes adversely affected the strength of the composites. This was different for the samples modified with diatomaceous earth alone, without silanes. In this case, the highest values were recorded for the systems containing beeswax. 

**Figure 7:**
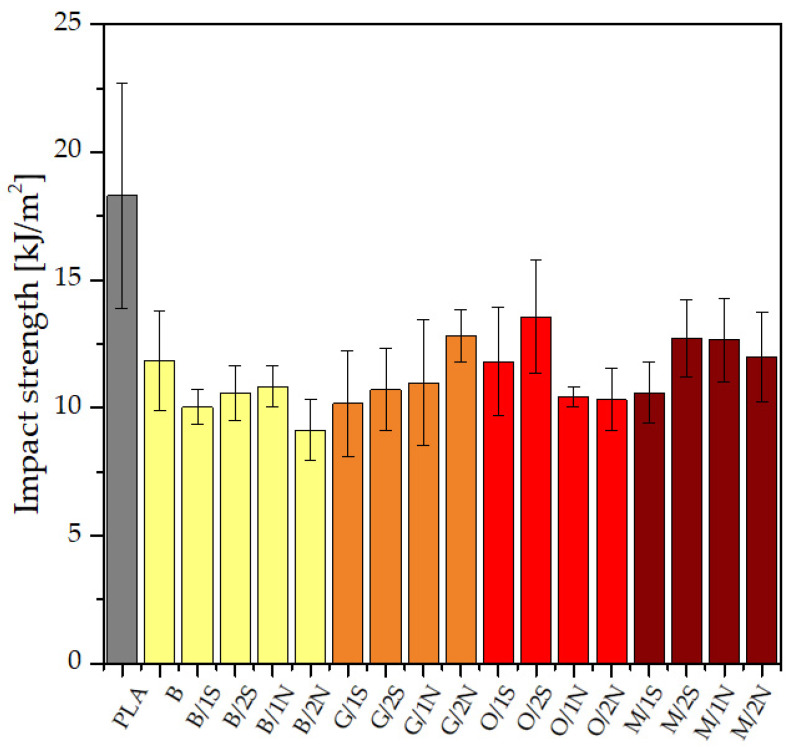
Impact strength of PLA matrix composites.

Neat PLA showed moderate impact resistance, while the standard deviation of the measurement series measurements was as high as about 5 kJ/m^2^, which may have been due to the low repeatability of the polymer crystallization (PLA is known for its low crystallization ability) during the process of obtaining the injection-molded specimens. The composites modified with diatomite earth showed slightly lower impact resistance values, but their standard deviations were significantly lower than that of the PLA, which may indicate the positive effect of diatomite on the processing of the PLA itself. The decrease in impact resistance when diatomaceous earth was introduced into the system was due, as previously mentioned, to the powder nature of the filler and its effect on the brittleness of the composite, especially at the relatively high fill tested for this paper (Figure 7). The addition of beeswax, depending on the silane used, resulted in an increase in impact resistance compared with analogous samples modified with synthetic wax. This effect was particularly evident for GPTMOS, and to a lesser extent for MTMOS. The opposite trend was observed for systems with OTES. In this case, the modification with synthetic wax caused a slight increase in this parameter, which indicated the incompatibility of the natural wax and the silane with a relatively long alkyl group, which was responsible for the formation of an interphase on the surface of the filler with a significantly increased hydrophobic character. This was evident in the MFR analysis as a large difference in the viscosity coefficient values for the O/xN and O/xS samples suggested that for these systems, the synthetic wax showed a higher wetting action on the filler action, creating a polymer/wax/filler interface, while the natural wax accumulated in a different phase and a polymer/filler interphase with a small proportion of wax was formed. This was also confirmed by static stretching, where the O/xS samples had a lower tensile strength than the O/xN samples due to the additional interphase reducing the polymer/filler interaction effect.

### 3.4. Thermal Tests

#### 3.4.1. DMTA

**Figure 8:**
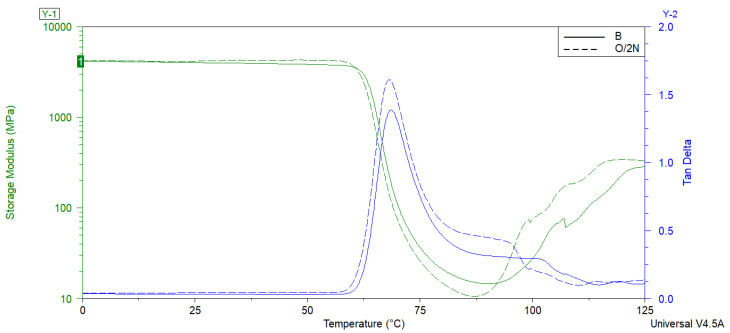
Representative results of the storage modulus and tan δ of the B and O/2N composites.

Table 3 and Figure 8 show how both the modification of diatomaceous earth with silanes and the addition of waxes influenced the storage modulus (G’) of the composites based on PLA and diatoms. For comparison, the results were collated with the glass transition temperature (T_g_) of the neat PLA. It can be observed that the modification led to the emergence of a T_g_ signal due to the nucleation effect, whereas for the neat PLA, no apparent relaxation was observed during heating, and only cold crystallization (T_cc_) onset at ~90 °C was visible as a small rise in E’.

**Figure 9:**
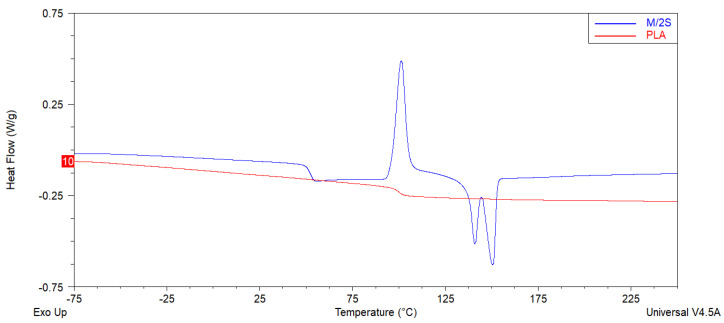
Representative DSC curves of unmodified PLA and M/2S composite obtained during second heating cycle.

The dynamic mechanical analysis allowed us to investigate the storage modulus (E’) and damping factor (tanδ) as a function of temperature. As can be seen in representative Figure 9, the temperature dependence of the storage modulus and damping factor for the PLA composites was similar. The obtained curves demonstrate a significant peak in the tan delta and a drop in the storage modulus at its narrow glass transition range. It should be noted that the storage moduli at 25 °C for the PLA composites with silane-treated diatomite were higher than the value for the neat PLA and the composites with untreated diatomaceous earth. Moreover, the relaxation intensities in the glass transition region of the composites containing silane were lowered and the peaks of the tanδ curves were shifted to lower temperatures compared with the PLA modified with untreated diatomaceous earth. This can be explained by the fact that the viscoelastic reaction of the polymeric chain was prevented by the formation of silane bonds. As a result, the glass transition temperatures were lowered gradually. These observations were confirmed for the PLA hybridized with epoxide-functionalized silane (GTMS) through reactive extrusion [26]. Looking into the curves, further G’ growth in the temperature range above 90 °C can be seen. This may have been caused by an increase in the mobility of the PLA macromolecules related to the cold crystallization of the PLA and its composites [27].

#### 3.4.2. DSC

A DSC analysis was performed to investigate the thermal behavior of the PLA and PLA/diatom composites. The results of the second heating cycle for the studied materials are collected in Table 4. With the temperature increasing in the 40–155 °C range, each DSC curve of the composites possessed three thermal characteristics: (a) a glass transition temperature (T_g_), (b) a cold crystallization event (T_cc_), and (c) an endothermic melting peak (T_m_) (Figure 9). The differences between the curves of the neat PLA and the composites are evident, as the curve of the neat PLA shows no apparent crystallinity or glass transition, and only a slight relaxation at ~90 °C, suggesting the onset of cold crystallization. The polymer, however, failed to undergo this event, probably due to a lack of nucleating agents and the heating rate applied in the experiment. This observation is in line with the DMTA analysis results, for which no glass transition was observed either, while a small local maximum of E’ was registered at ~90 °C. The addition of diatoms and silanes changed the thermal characteristics significantly. The composites showed a distinct glass transition temperature, an event of cold crystallization, and a melting event, where several endotherms were determined. Moreover, it was found that all the composites showed two distinct melting peaks (T_m1_ and T_m2_). The double melting peak was related to the recrystallization of the imperfect metastable crystals (T_m1_) and the melting of the perfected crystals (T_m2_) [28]. The application of silanized diatomite in combination with natural wax resulted in a reduction in T_cc_ temperature in comparison with the base diatomite composites, likely due to a reduction in melt viscosity and improved wetting action between the filler and the polymer, resulting in nucleation. In turn, however, the observed melting events occurred at lower temperatures, suggesting more crystal imperfections. On the other hand, silanization in combination with the application of synthetic wax, or the application of waxes in systems with base diatomite, showed that T_cc_ occurs at higher temperatures than in the PLA/base diatomite system, which results in the formation of both metastable and perfected PLA crystals with either fewer imperfections or larger diameters (higher T_m1_ and T_m2_, respectively). This was especially visible in the B/2N system and can be explained by the fact that the molten wax needed to be displaced from the diatomite surface before the nucleating effect occurred, while for the silanized diatomite, this effect took place at a lower temperature.

### 3.5. SEM Images of Composites

**Figure 10:**
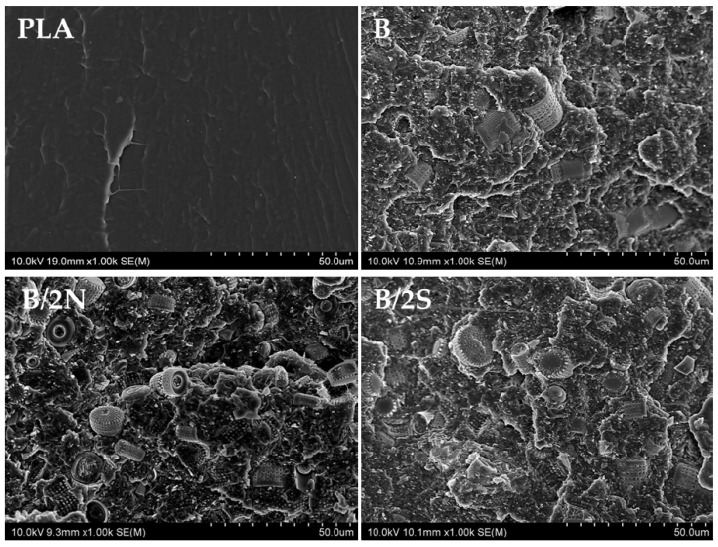
SEM images of fractured samples of composites without added silanes.

**Figure 11:**
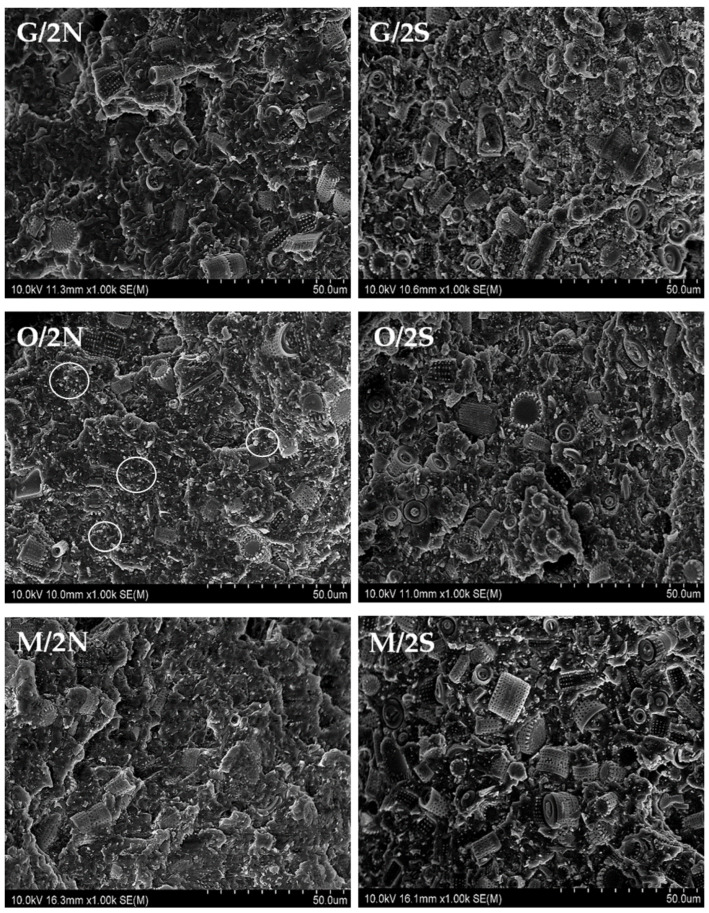
SEM images of fractured samples of selected modified composites.

**Figure 12:**
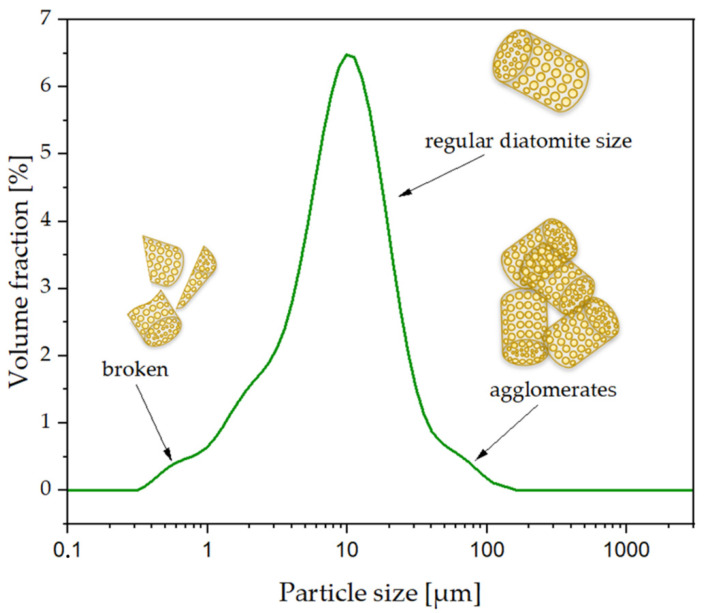
Particle size distribution of diatomaceous earth.

Using a scanning electron microscope, images of fractured samples of neat reference PLA and silane-free samples (Figure 10) and silane-modified composites (Figure 11) were taken. The images allowed us to assess the distribution of the diatomite particles in the PLA/DE composites. In the images taken of the composites modified with synthetic wax (2S), one can clearly see individual broken diatoms embedded in the polymer matrix. The PLA enters deep into the diatom frustule pores, which makes the connection permanent (the effect of the wetting action of the filler with the polymer). The use of a natural wax additive (2N) resulted, at least in part, in a lack of penetration of the PLA into the center of the diatom frustules. The filler particles appear to be on the surface of the polymer without being tightly bound to it. Moreover, in this case, a higher proportion of unbroken diatoms of smaller sizes (that is, in the range from 1 μm to a maximum of about 50 μm) is observed (Figure 12). It was not only the addition of various types of waxes that affected the surface image of the composites. The composites had particles of similar average size compared with the base diatomaceous earth, but did not tend to agglomerate, as seen in the SEM images taken. The number of diatoms in the systems was high, resulting in a large accumulation of diatoms, but they did not aggregate into large clusters. Despite the same filler concentration (25%) having been used, the fractures look different due to the silanization of the diatoms that was carried out before they were embedded in the composite. WTH-B synthetic wax caused increased agglomeration of the particles in the system, unlike natural wax, which caused significant ordering of the composite structure, i.e., individual diatoms are clearly visible and mostly have unbroken frustules. The differences between the silane-modified systems are significant. The aforementioned GPTMOS silane caused an even dispersion of diatomaceous earth in the PLA, similar to MTMOS silane, but with the addition of synthetic wax. In other cases, particle agglomeration occurred, and, in addition, the addition of beeswax to the O and M composites resulted in disordered structures in which there was a strong accumulation of the silanes used for the modification on the surface of the diatom frustules. Natural wax showed a lower wettability of the diatom surface due to its hydrophobic nature, so that the diatomaceous earth with smaller frustule size showed a lower dispersion in the polymer matrix, as seen in Figure 11 in the O/2N sample, among others.

**Figure 13:**
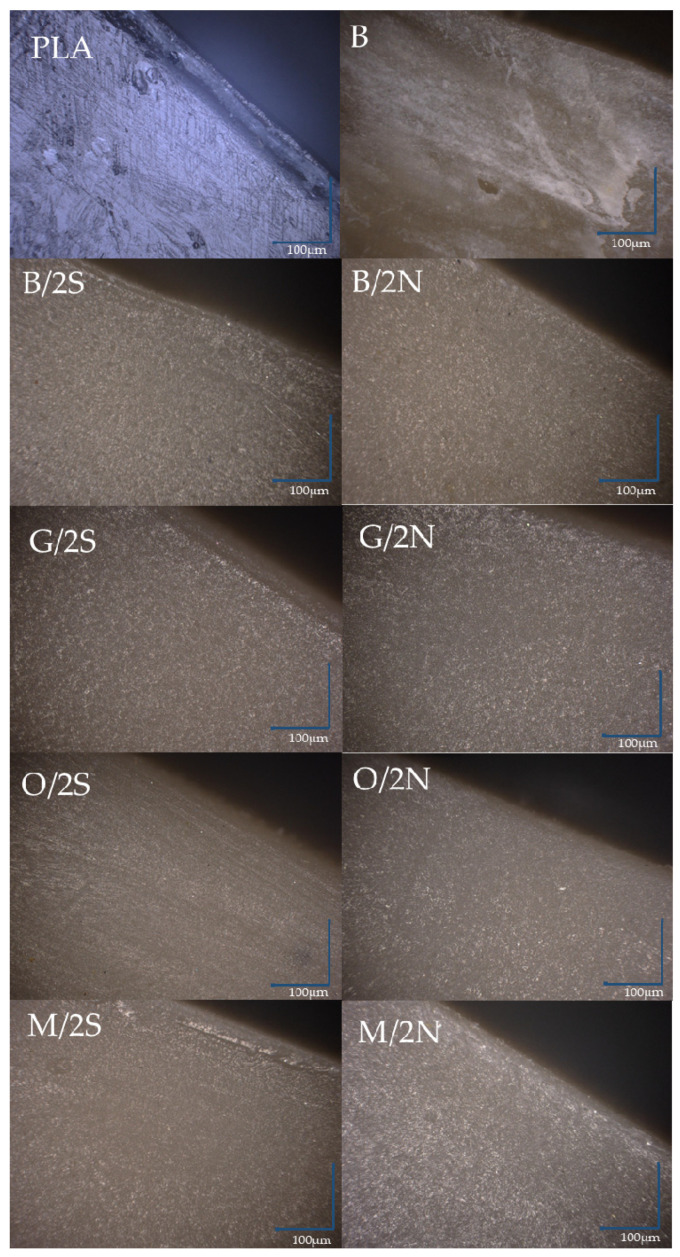
Light microscope images of the edges of PLA/DE composites.

Using an optical microscope, images were taken of the edges of the composites and the reference PLA (Figure 13). The neat PLA showed low scratch resistance, while no scratches or other defects were found on the surface of PLA/DE composites. The edge of the PLA, like the rest of the analyzed samples, was characterized by the presence of a thin layer with a different structure located near the surface. Depending on the filler and type of wax, its depth changed. The addition of the waxes alone resulted in a definite reduction compared with the composite containing only neat diatomaceous earth (B), in which surface discoloration occurred due to poor filler dispersion in the polymer matrix. The modification of the diatomaceous earth with silanes, especially GPTMOS silane, resulted in the formation of a free polymer layer on the sample surface. Considering the thickness of this layer, it can be concluded that natural (beeswax) wax causes this phenomenon to be inhibited, leading to the formation of a composite with a higher degree of dispersion. The composites filled with diatomaceous earth did not show the ability to transmit light, unlike the reference PLA. 

## 4. Conclusions

A series of diatomite-filled, PLA-based composites was fabricated, and the effects of the filler silanization and the addition of either natural or synthetic wax as processing aids was studied. For most of the systems studied, silanization did not affect mechanical properties of the composites in a significant fashion, proving either the presence of wax on the matrix–filler interphase or weak matrix–filler interaction, depending on the silane–wax system studied. It was observed by means of capillary viscosity, MFR, and DSC that there is an interaction between the waxes and the silanized surface of the filler, which impacts the wax segregation either in the filler–matrix interphase, or in the polymer matrix, which causes differences in melt rheology and thermal behavior, especially cold crystallization and melting. From the point of view of the processing and mechanical properties of the diatomite-filled composites, it is more advantageous to modify the PLA with silanized filler and synthetic wax than with untreated filler and/or no wax as this affords better control over the material’s processability. Synthetic wax has a positive effect on a number of composite properties, including mechanical properties, with a satisfactory level of tensile and flexural strength observed in particular, though this combination makes the produced composite less biodegradable and environmentally friendly than a composite containing beeswax. From the ecological point of view, natural waxes leave a smaller carbon footprint because of their production method. The selection of the appropriate wax to improve performance should be dictated by the desire to improve the specific properties of the composites, which vary depending on their type. Independently of the wax type used, satisfactory improvements in processing characteristics in terms of shear thinning rheology were obtained, and these are crucial for high throughput processing technologies, such as injection molding and extrusion. Synthetic wax, on the other hand, provided an increased melt flow index, making it a preferable choice for other processing methods, such as compression molding. 

## Figures and Tables

**Table 1:** Injection molding parameters.

Temperature (°C)	Nozzle	Zone 3	Zone 2	Zone 1	Feed
	190.0	195.0	200.0	185.0	40.0
Holding pressure		t (s)	0.0		9.0
		P (bar)	500.0		1500.0
Clamping force (kN)		Holding pressure time (s)	Cooling time (s)		Screw diameter (mm)
800		9.0	50.0		25.0

**Table 2:** The resulting compositions.

Full Name	Short Name
PLA	PLA
PLA/DE	B
PLA/DE + 1% ^(a)^ SW	B/1S
PLA/DE + 2% SW	B/2S
PLA/DE + 1% NW	B/1N
PLA/DE + 2% NW	B/2N
PLA/DE + 1% GPTMOS + 1% SW	G/1S
PLA/DE + 1% GPTMOS + 2% SW	G/2S
PLA/DE + 1% GPTMOS + 1% NW	G/1N
PLA/DE + 1% GPTMOS + 2% NW	G/2N
PLA/DE + 1% OTES + 1% SW	O/1S
PLA/DE + 1% OTES + 2% SW	O/2S
PLA/DE + 1% OTES + 1% NW	O/1N
PLA/DE + 1% OTES + 2% NW	O/2N
PLA/DE + 1% MTMOS + 1% SW	M/1S
PLA/DE + 1% MTMOS + 2% SW	M/2S
PLA/DE + 1% MTMOS + 1% NW	M/1N
PLA/DE + 1% MTMOS + 2% NW	M/2N

DE: diatomaceous earth; PLA/DE: PLA + 25% by weight; DE, SW: synthetic wax; NW: natural wax; GPTMOS: GPTMOS; OTES: OTES; MTMOS: MTMOS; ^(a)^: in all cases, additive % is given in weight-by-weight (*w*/*w*) ratio.

**Table 3:** Summary of the determined values of the glass transition temperature from storage modulus G’ curves.

Sample	T_g_ (°C)	Sample	T_g_ (°C)	Sample	T_g_ (°C)	Sample	T_g_ (°C)
PLA	N/A ^(a)^	–	–	–	–	–	–
B	61.6	–	–	–	–	–	–
B/2S	59.9	G/2S	58.5	O/2S	59.9	M/2S	59.0
B/2N	61.1	G/2N	57.7	O/2N	58.6	M/2N	60.0

^(a)^: T_g_ event not visible.

**Table 4:** Thermal characteristics of PLA and composites based on PLA with diatoms, silanes, and waxes.

Sample	Tg (°C)	Tcc (°C)	ΔHcc (J/g)	Tm_1_ (°C)	Tm_2_ (°C)	ΔHm (J/g)
PLA	N/A ^(a)^	–	–	–	–	–
B	46.1	99.9	25.3	131.0	142.4	24.8
B/2S	53.0	101.0	30.4	140.4	149.8	30.4
B/2N	53.4	109.9	28.8	143.5	151.9	29.3
G/2S	47.8	97.8	28.1	135.6	146.6	30.5
G/2N	42.1	92.9	24.3	124.8	137.7	24.7
O/2S	52.1	101.2	28.7	140.8	150.4	26.4
O/2N	43.5	93.4	24.3	128.1	140.0	26.4
M/2S	43.8	98.3	29.1	130.1	142.0	26.7
M/2N	41.0	92.5	21.4	122.6	136.7	23.6

^(a)^: Tg event not visible.

## Data Availability

Not applicable.
